# Algorithmic and human prediction of success in human collaboration from visual features

**DOI:** 10.1038/s41598-021-81145-3

**Published:** 2021-02-02

**Authors:** Martin Saveski, Edmond Awad, Iyad Rahwan, Manuel Cebrian

**Affiliations:** 1 Media Lab, Massachusetts Institute of Technology, Cambridge, MA USA; 2 Department of Economics, University of Exeter Business School, Exeter, UK; 3 Centre for Humans and Machines, Max-Planck Institute for Human Development, Berlin, Germany

**Keywords:** Human behaviour, Psychology

## Abstract

As groups are increasingly taking over individual experts in many tasks, it is ever more important to understand the determinants of group success. In this paper, we study the patterns of group success in Escape The Room, a physical adventure game in which a group is tasked with escaping a maze by collectively solving a series of puzzles. We investigate (1) the characteristics of successful groups, and (2) how accurately humans and machines can spot them from a group photo. The relationship between these two questions is based on the hypothesis that the characteristics of successful groups are encoded by features that can be spotted in their photo. We analyze >43K group photos (one photo per group) taken after groups have completed the game—from which all explicit performance-signaling information has been removed. First, we find that groups that are larger, older and more gender but less age diverse are significantly more likely to escape. Second, we compare humans and off-the-shelf machine learning algorithms at predicting whether a group escaped or not based on the completion photo. We find that individual guesses by humans achieve 58.3% accuracy, better than random, but worse than machines which display 71.6% accuracy. When humans are trained to guess by observing only four labeled photos, their accuracy increases to 64%. However, training humans on more labeled examples (eight or twelve) leads to a slight, but statistically insignificant improvement in accuracy (67.4%). Humans in the best training condition perform on par with two, but worse than three out of the five machine learning algorithms we evaluated. Our work illustrates the potentials and the limitations of machine learning systems in evaluating group performance and identifying success factors based on sparse visual cues.

## Introduction

Groups are increasingly taking over individual experts in the production of science, technology, public policy, art, strategic thinking and almost all aspects of human creativity^1–5^. Advanced communication technology allowing for global team coordination is speeding up this process^6–10^ to the point where labor economists conjecture that team management and composition may be the most critical skill to be learned in the near future^11–13^. As this process unfolds, we turn our attention to characterizing group performance and understanding how group composition predicts it.

We study the patterns of group performance in Escape The Room, a physical adventure game in which a group is locked in a room and has one hour to solve a series of interconnected puzzles (e.g., riddles, ciphers, symbol substitution with a key, assembly of a physical object, pattern identification, etc.) to find the key and escape the room. We are interested in answering the following research questions: (*a*) what are the characteristics of successful groups? and (*b*) how accurately can humans (non-experts) and machines (i.e., machine learning models) predict whether a group escaped or not? To answer these questions, we analyze photos of $>43\hbox {K}$ groups taken immediately after the game of groups across four locations in the U.S.

Focusing on Escape The Room presents a unique opportunity to study groups (1) performing a complex group task, (2) in a naturalistic setting, and (3) at scale.

(1): Traditionally studies that investigate group performance focus on short (10–15 min) and simple group tasks, such as, asking a group to brainstorm about possible uses of a brick, plan a fictitious shopping trip, or simultaneously type a text from a given hard copy. Escape The Room, on the other hand, is similar to many complex, real-world, tasks: it consists of many diverse and interconnected subtasks that cannot be simply distributed among the group members and solved individually, but require the group members to work together to solve them.
(2): To study group performance researchers usually invite participants in the lab, require them to fill a number of surveys (e.g., an IQ test), ask them—together with other strangers—to engage in a group task, and compensate them financially for their time. This is very different to how people encounter and face group tasks in the real world. Extracting information from Escape The Room group photos allows us to study groups in a naturalistic setting and to avoid any observer effects: the data we analyze was not initially captured for the purpose of the study and the individuals voluntarily participate in the task with others who are familiar to them.
(3): Lastly, previous studies have been limited to tens or hundreds groups. Here, we analyze the performance of $>43\hbox {K}$ groups over four locations in the U.S., captured over 1.5 years.

To achieve this we take a computational social science approach^14^ and apply a variety of computational techniques to answer the research questions of interest. We use computer vision algorithms for facial analysis to detect and extract the characteristics of the group members, and OCR (Optical Character Recognition) to analyze the signs that the group members hold in the photo to determine whether the group was successful (i.e., escaped the room). We apply machine learning algorithms to determine how accurately can machines predict group success and run Mechanical Turk studies involving hundreds of participants to determine how good are humans at spotting successful groups.

In summary, we make the following contributions:

- We collect photos of $>43\hbox {K}$ groups taken right after they have played Escape The Room. We use computer vision algorithms to extract detailed information about the group members and infer whether they escaped or not (“Data collection”).
- We study the relationship between the group characteristics and their likelihood to escape. We find that larger, older and more gender but less age diverse groups are more likely to escape (“Characteristics of successful groups”).
- We test how accurately can off-the-shelf machine learning algorithms predict whether a group escaped or not. We find that the best algorithms achieve an accuracy of 67% (“Machine predictions”).
- We also investigate how accurately can humans predict whether a group escaped or not. We find that, without any training, humans achieve an accuracy of 58%; however, training humans by showing them only 4 labeled photos significantly increases their prediction accuracy to 64%. Showing more training examples (8 or 12) leads to small, but not statistically significant improvements in accuracy (“Human predictions”).
- Finally, we compare the accuracy of the human and machine predictions on the same set of 1,000 photos. We find that untrained humans are less accurate than any of the machine learning algorithms. However, trained humans are as accurate as two, but slightly less accurate than three of the five off-the-shelf machine learning algorithms we considered (“Machine vs. human predictions”).

## Related work

This work falls under the intersection of three different topics: metrics of successful groups, the relation between facial appearance and personal characteristics, and the comparisons between humans and machines. In this section, we provide a brief review of the most relevant works in each of these areas.

### Factors for group success

Various studies have explored factors predicting group performance^15–19^. These factors include cognitive ability, cognitive diversity, social perceptiveness, gender diversity, group dynamics, and cultural diversity. Group performance is often measured in terms of performance in varying complex (online and face-to-face) tasks that measure creativity, innovation, and cognitive ability. One domain of group success that has been studied extensively is academia^1,20–23^. For example, AlShebli et al.^21^ investigated relations between academic success and five classes of diversity: ethnicity, gender, academic age, affiliation, and disciplinary. They found that affiliation diversity and disciplinary diversity are the weakest predictors, while ethnic diversity has a strong correlation with success. Further, they established a causal link between ethnic diversity and academic success.

### Visual cues as signals

Beliefs that facial appearance can provide clues to the nature of the mind and personality can be dated back to ancient societies^24^. Recent studies explored this relation in terms of trustworthiness^25–27^, aggressiveness^28,29^, and electoral success^30,31^. For example, Oosterhof and Todorov^25^ showed that judgments of faces, can be approximately characterized by judgments of trustworthiness and dominance, and Bonnefon et al.^27^ showed that trustworthiness detection from visual cues is an encapsulated, automatic process, where the observer has little insight at the conscious level. Recent studies have shown that the facial appearance in profile photos of individuals who frequent a place (e.g., restaurant) can be used to predict the ambiance of the place^32,33^.

### Human vs. machine performance

Recent advances in technology has been evaluated by showcasing machines outperforming humans in various complex games that had been long considered as indicators of human intelligence^34–38^. Identifying a successful group based on a photo presents a different challenge as it requires both measuring the group characteristics using computer vision capabilities and modeling the relationship between the group characteristics and success.

## Data collection

**Figure 1:**
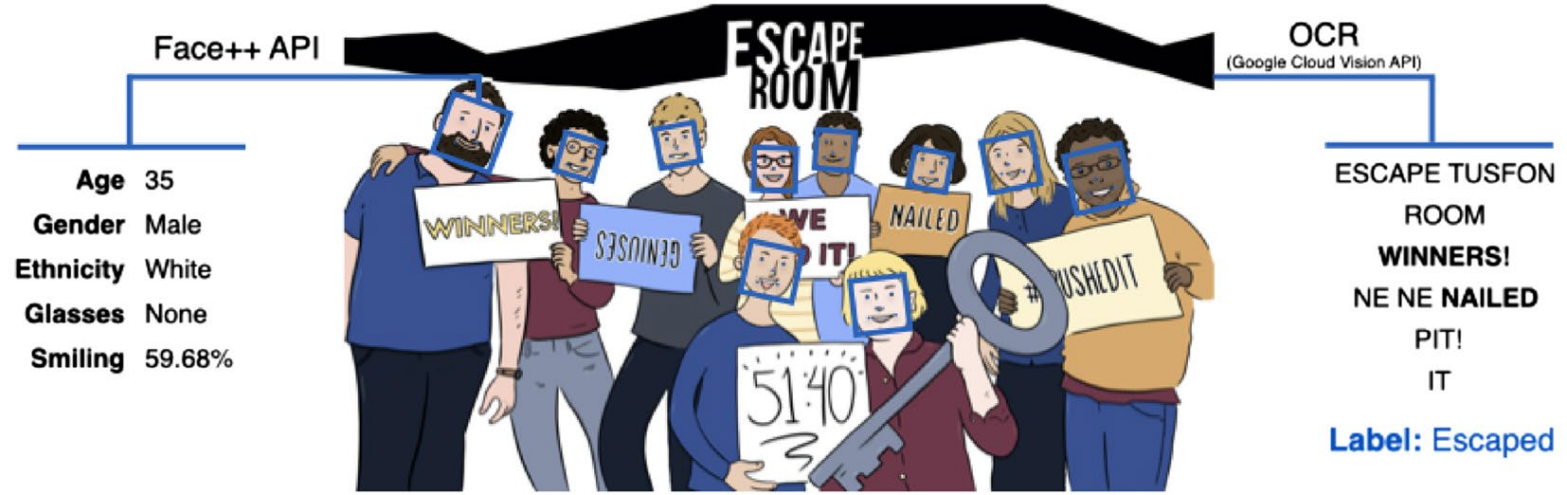
A sketch of a real photo of a group after playing Escape The Room. We used Face++ to locate and analyze the faces of the group members. The rectangles show the locations of the detected faces. The annotation on the left shows the features extracted for the group member on the far left. To determine whether the group escaped or not, we used OCR to extract the text on the signs that the participants hold (raw OCR output shown on the right of the photo) and checked for keywords that appear on winning or losing signs. In this example, two winning keywords were detected (in bold) and the photo was labeled as escaped. All data analysis and experiments presented in this paper were done on real photos. We use this illustration only to describe the process.

We collected data from the public Facebook pages of four Escape The Room venues. In each venue, after playing the game, groups are offered to take a photo holding entertaining signs: if they escaped they hold signs that say, e.g., “winners!”, “geniuses”, “nailed it!”, and a sign that shows the time it took them to escape; if they did not escape, they hold signs that say “losers”, “help us!”, “so close...” (Fig. 1). In both cases, the photos are uploaded to the public Facebook pages of the Escape The Room venues.

We collected data from four Escape The Room venues: New York City, Boston, Houston (Texas), and Scottsdale (Arizona). At the time of the data collection all venues had been active for about 1.5 years, except for the New York City venue which has been operating for about 2.5 years.

We used the Facebook Graph API to collect the URLs and then download all photos posted on these pages. If available, we also captured the name of the “room”, i.e., the particular puzzle that the group played. We collected a total of 49,894 photos—NYC: 27,674; Boston: 8,089; Scottsdale: 8,663, Houston: 5,468.

### Feature extraction

To determine the composition of the groups, we applied computer vision algorithms. We used Face++ (https://www.faceplusplus.com), a software based on deep learning, specialized for face recognition and facial analysis that has been demonstrated to have an excellent accuracy on these tasks^39^.

We detected the faces on each photo and extracted the location (*x* and *y* coordinates of the rectangle containing the face), demographic and other characteristics of each face, including: gender (male or female), age, ethnicity (Asian, Black, or White), smile index (a real number between 0 and 100; 0: no smile, 100: grin), and whether the subject wears glasses (none, normal, dark).

### Labeling

To determine whether a group in a photo escaped or not, we exploited the fact that groups that escaped hold different signs than those that did not. We compiled two lists of words: one of words that appear exclusively on the winning signs, and another of words that appear exclusively on the losing signs. Next, we used Google’s Cloud Vision API to run OCR (Optical Character Recognition) and extract the text that appears in the photos. Then, we checked whether any of the words in the two lists (escaped vs. did not escape) appear as a substring in the extracted text; if we found at least one word from one of the lists, then we labeled the photo correspondingly.

In the majority of the photos ($64.3\%$), we found more than one non-conflicting keyword in the extracted text, which reassured us in the validity of this automated labeling method. In $22.2\%$ of the photos, we found just one keyword. We discarded $13.6\%$ of the photos for which we did not find any keywords, or we found conflicting keywords (both success and non-success words). Thus, we ended up with 43,113 labeled photos, i.e., $86.4\%$ of all photos. We find that 18,001 out of 43,113 photos were of groups that escaped, for an overall success rate of $41.75\%$. To further validate the labels, we manually looked at a random sample of 500 photos and found only one mislabeled photo.

## Characteristics of successful groups

Next, we look for associations between the characteristics of the group and its likelihood to escape the room.

### Features

To describe the groups, we compute aggregate statistics of the faces detected in the photos and we create the following features:

- *Number of people.* The total number of faces detected in the photo. We expect larger groups to have an advantage, since the game is the same regardless of the group size. However, the group size may influence the group dynamics, e.g., larger groups may require more coordination, which in turn may deteriorate the group performance.
- *Gender diversity.* To quantify the gender diversity of the group we compute the Shannon’s entropy of the gender distribution. Shannon’s entropy is a standard approach to measuring diversity, grounded in information theory.
- *Ethnic diversity.* We measure the ethnic diversity of the group as the Shannon’s entropy of the ethnic distribution, i.e., the breakdown of White, Asian, or Black individuals in the group. The set of ethnicities we consider is constrained by the output of the Face++ API.
- *Age (mean and standard deviation).* To quantify the overall age and the age diversity of the group, we compute the mean and the standard deviation of the (estimated) age of the group members.
- *Physical proximity of the group members.* We quantify the physical closeness between the group members as follows: (1) we build a fully connected graph where each node represents a person and the edges between the nodes are weighted by the Euclidean distance between the corresponding faces on the photo; (2) we compute the minimum spanning tree of the graph, (3) we compute the ratio of the sum of the edge weights in the minimum spanning tree and the number of people in the group. We expect that group members who are more familiar with each other to feel more comfortable to be closer together when posing for the photo.
- *Smiling index (mean and standard deviation).* To capture the overall emotional expression of the group and the variation of the emotional expressions of the group members, we compute the mean and the standard deviation of the (estimated) smiling coefficients of the group members. The smiling coefficients range from 0 (no smile) to 100 (grin).
- *Fraction of group members wearing glasses.* We compute the fraction of group members that wear normal (not dark) glasses.

**Figure 2:**
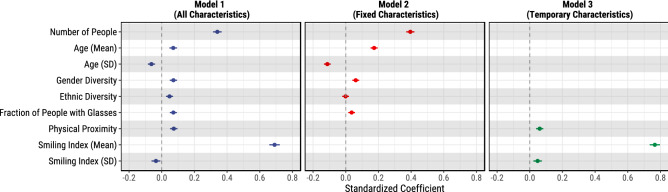
Coefficient plots of logistic regression models using *escaped?* (binary variable indicating whether the group escaped) as a dependent variable and the group characteristics as independent variables. The error bars represent 95% confidence intervals.

### Analysis

Next, we run a series of logistic regression models using *escaped?* (binary, yes/no, variable) as a dependent variable and the features described above, or a subset of them, as independent variables (Fig. 2). We also control for location (rooms in different locations may be slightly differently arranged due to space constraints) and room type (escaping some room types may require solving more or harder puzzles) effects.

Some group characteristics, such as group size, age distribution, gender and ethnic diversity, are *fixed*, i.e., they are the same before and after the group performed the task. While other characteristics, e.g., the physical distance between the members of the group photo or the distribution of the smiling indices, are *temporary* and likely different in response to the performance of the group. Therefore, we test three model formulations: *Model 1*, using all group characteristics; *Model 2*, using only the fixed characteristics; and *Model 3*, using only the temporary characteristics. We observe the same patterns in the full model (Model 1) and the models that include a subset of the characteristics, for all except two characteristics: (1) the coefficient for ethnic diversity becomes statistically insignificant when we consider only fixed characteristics, and (2) the sign of the smiling index standard deviation coefficient switches from negative to positive when we consider only the temporary characteristics. We proceed by reporting the results of the full model (Model 1).

To illustrate the results, we report predicted values, e.g., how the probability that a group will escape changes as we vary one feature and fix all other features to their mean values. We use simulations ($n=10^7$) to account for the fundamental and estimation uncertainty of the model^40^.

We find that larger groups are significantly more likely to escape. Other things being equal, a group with 5 members has a probability to escape equal to 0.34, whereas a group of 10 has a probability of 0.52.

Gender and ethnic diversity play a role as well. Groups with higher diversity, in both gender and ethnicity, tend to be more likely to escape. For instance, a group with no gender diversity (all females or all males) has a probability to escape of 0.39, whereas a perfectly balanced group has a probability of 0.44. Similarly, ethnically homogeneous groups (entropy = 0) have lower probability to escape: 0.42, compared to perfectly heterogeneous groups (entropy = 1) which have a probability of 0.47. We note, however, that the effect of ethnic diversity becomes insignificant when we consider only the fixed characteristics.

When it comes to age, older groups tend to be more likely to escape: other things being equal, a group with an average age of 20 will escape with a probability of 0.41, whereas a group with an average age of 40 has a probability of 0.47. However, age diversity makes groups less likely to escape. Perhaps, age differences between the group members change the dynamics of the group.

Groups in which the group members are physically closer to each other on the photo are also more likely to escape. Similarly, groups in which higher fraction of members wear glasses are more likely to escape. For instance, other things being equal, a group in which none of the members wears glasses has a probability to escape of 0.41, whereas a group in which half of the members wear glasses has a probability of 0.47.

Finally, as one might expect, groups that smile on the photo taken after the game (i.e., have higher mean smiling index) are more likely to have escaped. For example, a group with mean smiling index of 60 (slightly below the mean) has a probability to escape of 0.36, whereas a group with a mean smiling index of 80 (slightly above the mean) has a probably to escape of 0.54.

In summary, larger and more gender diverse groups are more likely to escape. Also, older but less age diverse groups tend to be more successful. Groups with more members that are smiley and wear glasses were more likely to have escaped.

## Machine predictions

Next, we investigate how accurately machine learning methods can predict whether a group has escaped or not. We note that since the photos were taken after the groups have completed the game, we use prediction to refer to the process of guessing whether a group was successful based on its characteristics in the absence of information about the outcome, rather than forecasting, i.e., predicting future events.

### Extended feature set

Since the goal of this experiment is only prediction, not interpretation as in the previous section, we expand the feature set. In addition to the features described above, we include the following features: (1) counts for each gender value (males, females, and unidentified), (2) counts for each ethnicity value (Asian, White, Black, and unidentified), and (3) counts for each *glasses* value (normal glasses, dark glasses, no glasses, and unidentified). Resulting in a total of 30 features. We experimented with scaling the features such that every feature has zero mean and unit variance, but we did not observe any significant improvements.

### Classifiers

We test five off-the-shelf machine learning algorithms: (1) Logistic Regression, (2) Naive Bayes, (3) Linear SVM, (4) Random Forest, and (5) Gradient Boosted Regression Trees. We tuned the algorithms that have hyper-parameters by testing a number of different values: Linear SVM, $c \in \{0.01, 0.1, 1.0, 10\}$; Random Forest and Gradient Boosted Regression Trees, number of estimators $\in \{10, 100, 1000, 10000\}$. As a baseline we use a dummy classifier which always predicts the most common class in the training data.

### Evaluation

To evaluate the algorithms, we ran nested 10-fold cross-validation^41^. In the outer loop, we divided the full dataset in 10 partitions: in each iteration we used 9 partitions as a development set and the remaining one as a test set, repeating this process 10 times using each partition as a test set only once. In the inner loop, we performed 10-fold cross-validation on each development set to evaluate the different hyper-parameters and then trained the algorithm on the full development set using the best hyper-parameter settings. To create the partitions, both in the inner and outer cross-validation loops, we took stratified samples, preserving the same ratio of escaped vs. not escaped photos as in the full dataset. To avoid differences due to the randomness in the partitioning of the data, we evaluated all algorithms on the same partitions. To ease interpretation of the results, we used accuracy as an evaluation metric. We verified that measuring AUC (Area Under the ROC Curve) instead of accuracy leads to the same ranking of algorithms.

**Figure 3:**
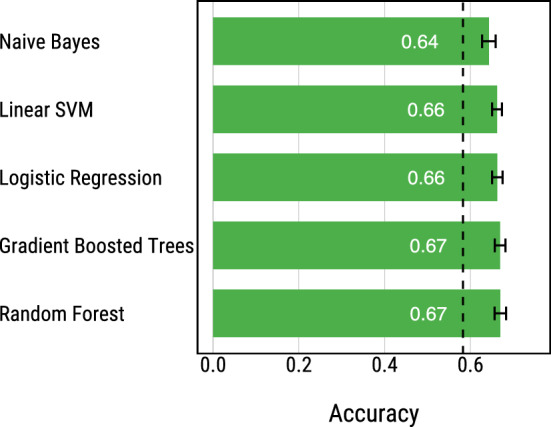
Accuracy of five off-the-shelf machine learning algorithms at predicting whether a group will escape the room computed using nested 10-fold cross-validation. The error bars show 95% confidence intervals computed over the 10-folds of the data. The dashed black line shows the accuracy of a dummy classifier that always predicts the most frequent class in the training data.

### Results

We find that all classifiers outperform the baseline classifier that predicts the most common class (Fig. 3). Random Forests and Gradient Boosted Regression Trees perform best with accuracy of 66.99% and 66.92%, respectively. (The difference is statistically indistinguishable, Wilcoxon signed-rank test^42^, $p = 0.54$). Logistic Regression and Linear SVM perform slightly worse, achieving accuracy of 66.26% and 66.18%, respectively (this difference is also statistically indistinguishable: Wilcoxon signed-rank test, $p = 0.26$). Finally, Naive Bayes achieves the worst performance with accuracy of 64.3%.

## Human predictions

**Figure 4:**
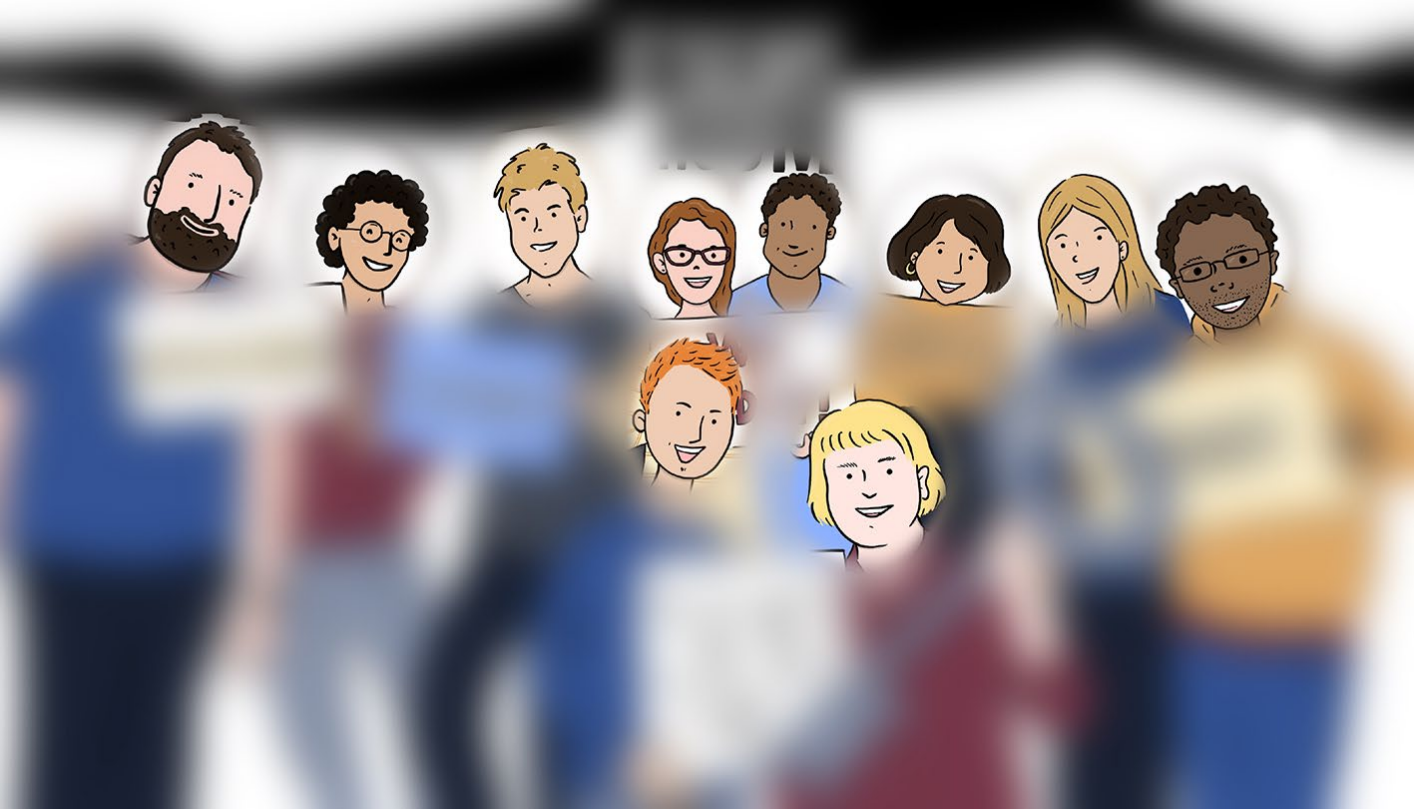
A sketch of a real photo shown to study participants asked to predict whether a group escaped or not (blurred version of the photo in Fig. 1). All performance-signaling information has been obfuscated, while still allowing participants to see the body posture and physical proximity of the group members.

Next, we turn to measuring the human accuracy at predicting whether a group escaped or not. We blur the photos to obfuscate any explicit performance signaling information (e.g., the signs), but keep the faces of the group members clear (Fig. 4). We also made sure that the photos are not too blurred so that study participants can see the body posture and the physical proximity of the group members. We run two studies. In the first study, we measure the baseline accuracy of the participants’ predictions and test whether training participants by showing them only four labeled photos improves their accuracy. In the second study, we investigate whether showing participants four training examples is enough and whether showing more labeled photos significantly improves their accuracy.

### Study 1

The goals of this study are to measure the baseline accuracy of the human predictions and to test whether training participants by showing them a sample of labeled photos will improve their prediction accuracy.

#### Experimental setup

For the purpose of the study, we used a random sample of 2,000 photos extracted from the full dataset. We recruited 400 participants (U.S. residents only) using the Mechanical Turk platform for a compensation of 60 cents per participant.

We randomly assigned participants to one of two conditions: *training* (treatment) and *no training* (control). Participants in both groups went through a “warm up” phase. However, in the *training* (treatment) condition participants were first shown four blurred photos, asked to predict whether the group on the photo escaped or not, and then were given the answer, i.e., shown the original (unblurred) photo. The photos were shown one at a time, giving participants immediate feedback before the next prediction. In the *no training* (control) condition, participants went through the same process, but they were not shown the original, unblurred photos. Participants in both conditions were told that this stage is only meant to help them familiarize themselves with the task and that their answers will not be taken into account.

After the initial training stage, we gave participants in both conditions the main task. We showed each participant 10 blurred photos (similar to Fig. 4) and we asked them to predict whether the group shown in each photo escaped or not. To avoid confusion, we showed participants one photo at a time.

To ensure that the participants in the two conditions were shown the exact same set of photos, we randomly assigned each photo to one and only one participant in each condition. The participants made a total of 4,000 predictions (400 participants, 10 predictions / participant) and each photo was rated by exactly two participants, one from each condition.

#### Results

**Figure 5:**
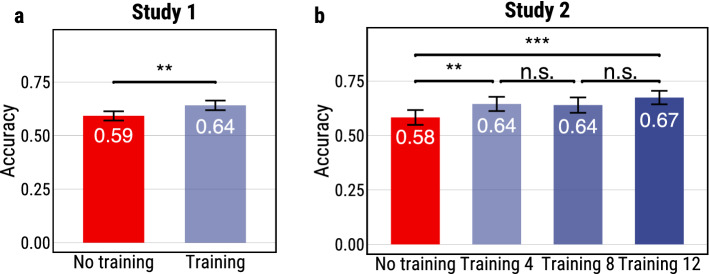
**(a)** Results of Study 1. Comparison between the prediction accuracy of participants in the two conditions: no training (control) vs. training (treatment). Trained participants, who were shown four labeled photos, perform significantly better than untrained participants. **(b)** Results of Study 2. Comparison between the prediction accuracy of participants who were not shown any training photos and participants who were shown 4, 8, or 12 training photos. As in Study 1, showing participants four labeled photos significantly improves their prediction accuracy. However, the improvements of showing additional labeled photos are not statistically significant. (The error bars represent $95\%$ confidence intervals; ***$p<0.001$, **$p<0.01$, *$p<0.05$).

To compare the accuracy of the participants’ predictions in the two conditions, we first compute the accuracy of each participant on the ten photos they were shown and then compute the mean accuracy of all the participants in the same condition. We find that training increases the accuracy of the participants’ predictions by 5% (Fig. 5a), a statistically significant increase ($p= 0.002$). Trained participants achieve a mean accuracy of 64% (95% CIs [62%, 66%]) superior to the accuracy of the untrained participants with a mean accuracy of 59% (95% CIs [57%, 61%]).

It is worth noting that since participants in both conditions were shown the same four blurred photos before being asked to performed the main task, we know that the improved accuracy of the trained participants is not due to mere exposure to more blurred photos or more familiarity with the blurred photos. Instead, the difference in accuracy must be due to exposure to ground truth (i.e., original photos), a process similar to the training of machine learning models.

### Study 2

In Study 1, we found that training participants by showing them four labeled photos significantly improves their performance compared to no training at all. This prompts the question of whether participants will make even more accurate predictions if they went through a longer training phase and were shown more labeled photos. The answer to this question is non-obvious and may unfold in three different ways: (1) by seeing more labeled photos participants may have a better chance at detecting more nuanced patterns in the photos of successful vs. unsuccessful groups and further improve their performance, (2) participants may have already inferred all important patterns of success and longer training phase will not have any effect on their accuracy, or (3) longer training phase may lead to respondents fatigue and deteriorate their prediction accuracy. In this study, we run another experiment to answer this question empirically.

#### Experimental setup

We randomly assigned participants to one of four conditions: *no training* (control), *training with 4*, *training with 8*, or *training with 12* photos. We recruited 400 participants (100 per condition) from the the U.S., prohibiting participants who took part in Study 1 to participate again. We increased the compensation rate to $1 as we expected the task to take significantly longer due to the longer training phase in two of the four conditions.

In the training phase, we used a random sample of 1,200 photos from the full dataset. To ensure that the training photos are as similar as possible across different conditions, we selected them in batches. In each batch, we first took a batch of 12 from the 1,200 photos and used these photos in the *training 12* condition. Then we took a subsample of 8 and 4 photos out of these 12 and used them in the *training 8* and *training 4* conditions, respectively. We repeated this process 100 times. In all cases, we ensured that the samples contain an equal number of positive and negative examples. To select the photos for the testing phase, we randomly sampled a different group of 1,000 photos and then divided the 1,000 photos into groups of 10 and used the same groups across all four conditions. Photos shown in the training phase did not appear in the test phase.

The task was exactly the same as in Study 1. In the training phase participants were shown a blurred photo, asked to guess whether the group escaped or not, then shown the original photo, repeating this process 4, 8, or 12 times depending on the condition they were assigned to. Participants in the no training condition skipped this phase. In the test phase, participants were shown only blurred photos, asked to guess whether the group escaped and to indicate their confidence. Participants in all conditions were shown 10 test photos.

#### Results

Similar to Study 1, we compute the accuracy of each participant over the 10 test photos and then we compute the mean accuracy of the participants over the four different conditions.

First, we observe that our findings from Study 1 are replicated in this study: training participants by giving them feedback on their predictions on only four training photos significantly increases their accuracy over no training ($$p=0.01$$). Beyond replicating the same pattern, we observe very similar levels of accuracy in the two conditions across the two studies: no training, 58.2% accuracy; and training on four photos, 64.5% accuracy (Fig. 5b). This also suggests that the increase of the compensation from 60 cents to $1 did not have a significant effect on the accuracy.

Second, we find that the returns of training participants on more photos are diminishing. Training participants on 8 instead of 4 photos leads to almost the same level of accuracy, 63.96% ($p=0.84$). Similarly training participants on 12 instead of 4 photos leads to accuracy of 67.4% vs. 64.5%, The improvement of 2.9% is not statistically significant ($p=0.2$).

### Human predictions and group characteristics

Besides asking participants of the studies to make predictions, we also asked them to explain how they made their decisions. At the end of the survey, participants were given a list of group characteristics and asked to report which, if any, of the listed characteristics they considered while making their predictions (they could select multiple characteristics).

**Figure 6:**
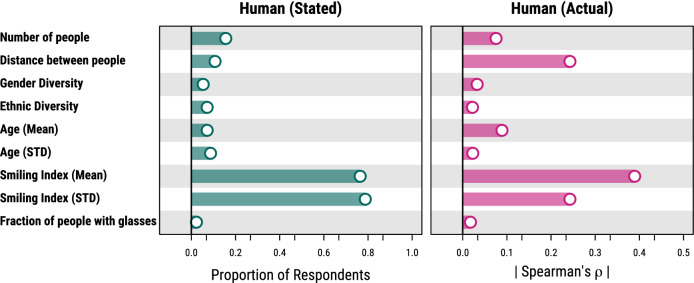
Analysis of the group characteristics used by the participants in Study 1 to make their predictions. The left panel shows the “stated” relevance, i.e., how often each group characteristic has been reported as important by the participants. The right panel shows the “actual” relevance, i.e., absolute value of the correlation (Spearman correlation) between the predictions made by participants (escaped or not) and each of the nine characteristics.

Here we look at which group characteristics participants used to make their predictions. We compute two metrics: (1) the fraction of participants who reported that a given group characteristic was useful in making their prediction, and (2) the actual correlation between the participants’ predictions and the group characteristics (Fig. 6). Since the direction of the correlation is irrelevant, to ease the comparison, we report the absolute values of the correlations. While the first metric captures the group characteristics that participants were aware of, the second captures the group characteristics that actually influenced participants the most.

We find that the overall emotional expression of the group members (i.e., mean smiling index) is by far the most used group characteristic, both according to the participants reports and the correlation with their predictions (Fig. 6). We also find that participants tend to overestimate the importance of the diversity of emotional expressions of the group members (i.e., standard deviation of the smiling index), but underestimate the importance of the physical distance between the group members and the overall age of the group. The last two, although more correlated to the participants’ predictions, are less often reported as important characteristics by the participants. This suggests that these characteristics may have subconsciously influenced the participants’ predictions.

## Machine vs. human predictions

Next, we compare the accuracy and the alignment between the human (trained and untrained) predictions and the predictions of the machine learning algorithms.

To build the machine learning models, we used as a development set all photos in the dataset except for the 1,000 test photos that were shown to participants in Study 2. We performed 10-fold cross-validation to tune the hyper-parameters (considering the values listed in “Machine predictions”) and then we trained the models on the full development set using the best hyper-parameter settings. (This corresponds to a single inner loop of the nested cross-validation we performed in “Machine predictions”). Then, we tested the models on the 1,000 test photos rated by the participants in Study 2 and compared the accuracy of the human (trained and untrained) and machine predictions. Note that due to the difference in the experimental setup these results are slightly different from the ones reported in Fig. 3 where we ran nested 10-fold cross-validation.

**Figure 7:**
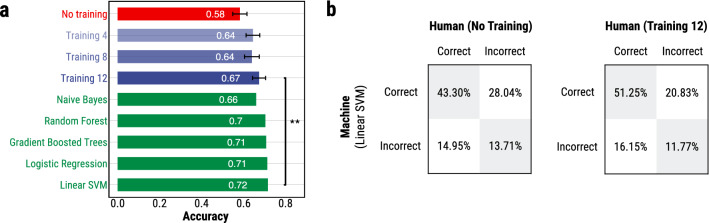
Results of Study 2. **(a)** Comparison between the accuracy of human (across the four different conditions) and machine learning predictions on a random sample of 1,000 photos. The machine learning algorithms were trained on the remaining 42,113 photos from the full dataset. The error bars on the human accuracy represent 95% confidence intervals. **(b)** Comparison of the correctness of the predictions of the most accurate machine learning model (Linear SVM) and the predictions of the untrained (left) and best trained (right) participants. In both cases, there is a significant proportion of photos (42.99% and 36.98%, respectively) where only the human or only the machine predictions are correct. (**$p<0.01$).

We find that the predictions of the untrained participants are consistently less accurate than all of the machine learning models by a margin of 8–14% (Fig. 7a). However, trained participants in the best performing condition (Training 12) perform on par with some of the algorithms, Naive Bayes ($p=0.369$, testing the null hypothesis that the mean accuracy of the participants is equal to the accuracy of the model) and Random Forest ($p=0.063$), but worse than Gradient Boosted Regression Trees ($p=0.036$), Logistic Regression ($p=0.017$), and Linear SVM ($p=0.009$) by a small margin of 3–4% (Fig. 7a). We note that all algorithms have higher accuracy on the 1,000 test photos than their mean accuracy measured using nested 10-fold cross-validation on the full dataset (Fig. 3). In fact, the accuracy of the participants in the *training 12* condition (67%) is the same as the accuracy of the best performing machine learning algorithms (Gradient Boosted Regression Trees and Random Forest) when evaluated on the full dataset (Fig. 3).

We compare the correctness of the predictions of trained and untrained participants and the best machine learning model (Linear SVM). We find that there is a significant proportion (more then one third) of cases where only the human prediction or only the machine predictions are correct (Fig. 7b). This is the case both for untrained ($28.04\% + 14.95\% = 42.99\%$) and trained ($20.83\% + 16.15\% = 36.98\%$) participants.

We run a series of logistic regression models using a number of boolean indicators—(1) whether both machine and human predictions are correct, (2) whether both are incorrect, (3) whether only the machine prediction is correct, and (4) whether only the human prediction is correct—to characterize the difference in predictions. We find that both humans and machines tend to be misled by smiley groups; however, machines are better at identifying other group characteristics such as the importance of the group size and gender diversity.

This naturally leads to the question of how human and machine predictions can be combined to achieve greater accuracy than each of them individually.

## Discussion

### Summary

We studied which group characteristics are associated with success and tested how well humans and machines can spot successful groups based on their photos. We analyzed $>43\hbox {K}$ photos of groups who had played Escape The Room. Our findings identify group characteristics that correlate with their success, establish baseline performance of humans and off-the-shelf machine learning algorithms in this task, and show that humans can increase their prediction success by training. Our results on factors predicting group success contribute to the vast literature showing converging evidence of which factors that describe groups and their members are associated with their success^15,17–19^.

Why does the prediction work and what does it mean? Our hypothesis is based on the literature that shows the importance of individuals’ competence level, the composition of individual characteristics, and their synergies^15,17,18^. These factors are encoded by features that are visible in a group photo. For example, the average age of the group can be a proxy for their life experience, while the variance of group age can correlate with the synergy among their members (i.e., higher synergy for small age variance). Temporary visible cues of groups such as the physical proximity of their members, and their visible emotional states (e.g., smiling) can provide another signal on their success. While it is hard to characterize the causal links between these factors and the success of a group (and thus it is hard to draw ultimate inferences on why they exist), they are clearly relevant to the prediction of its success.

### Limitations

Being able to study tens of thousands of groups gives us a unique opportunity to advance our understanding of group dynamics. Nevertheless, our study suffers from four main limitations. First, we exclusively studied groups that played Escape The Room, only one out of many collaborative problem-solving tasks. While we believe that Escape The Room has common features with many other complex tasks that consist of multiple interconnected subtasks, we would like to warrant against blindly extending the conclusions of our findings to other tasks. The generalizability of our results is dependent on (1) what group characteristics and features can be recovered by the use of their photo and (2) on the complex relationship between these features and the task at hand (in this case, Escape The Room). The former is fairly generalizable to any group photo, and many of the group characteristics we measured in “Characteristics of successful groups” (e.g., group size, age, gender, and ethnic diversity) can be relevant in other scenarios. The latter is harder to assess; the complex relationship between these features and success will ultimately be influenced by the nature of the task. However, we believe that the factors of success (e.g., average age or gender diversity) revealed by our analysis are convincing that this relationship is sensible in this context, and so their generalizability to other tasks can be qualitatively hypothesized as a first step.

Second, our sample of groups that played Escape The Room consists of people who (1) chose to play this game, (2) chose to play in the same group, and (3) chose to take a photo after finishing the game. Each of these choices adds a limitation to the representativeness of this sample. First, we define the population of interest as people who are interested in working collaboratively in a group to solve problems. We argue that this is a realistic choice of population since it describes many real-life work environments. However, it excludes people who have to accept working in a group as part of their job. Second, we look at how each of the three choices affect the representativeness of our sample. As per (1), it is possible that there exists a subset of people who are interested in working in groups, yet are undersampled (or non-existent) in our data (after all, choosing to participate in Escape The Room requires interest in games and adventure). In this case, our sample may be more representative of a slightly different group of people; those who are interested in working *and playing* collaboratively in groups to solve problems *as part of an adventure game*. As per (2), while in many settings, including at work, many people do not get to choose the group of people they will work with, it is reasonable to suggest that groups who played in Escape The Room might have been formed in these settings, e.g., a group of work colleagues or people who met in a business or an academic setting, became friends, and went on to play the game together. As for (3), it is reasonable to expect that a group that avoids taking a photo together is more likely to have failed to escape. So, it is possible that unsuccessful groups are underrepresented in our sample. Nevertheless, we believe that most groups did take a photo, given that all groups are offered to take a photo at the end of the game.

Third, an intrinsic limitation of this study is that the photos that we analyzed were taken after the groups have completed the game. To take this into account in our analyses of the characteristics of successful groups (“Characteristics of successful groups”), we split the group characteristics into fixed (group size, age, gender, and ethnic diversity) and temporary (physical proximity and smiling index), and studied their relationship with success separately and together (Fig. 2). However, in our comparison of how well humans and machines can predict group success (“Machine predictions”, “Human predictions”, and “Machine vs. human predictions”), we could not separate the two types of group characteristics as the photos we showed to the study participants conveyed both the groups’ fixed and temporary characteristics. Future studies that have access to the groups’ temporary characteristics before the task can analyze how situational factors right before the task, such as the mood and the physical proximity of the group members, influence group success.

Fourth, one of the key challenges in comparing the prediction accuracy of humans and machines was to level the playing field. The two have very different advantages. Humans have a life-long experience directly participating in group activities, observing and evaluating groups in the real world and in simulated environments such as in movies or television. Machines, on the other hand, are tireless at processing large amounts of data—examining 42K photos would be a tedious, and uninteresting task for any individual. To ensure a fair comparison, we took a number of steps: (1) we trained the models using only features that encode information that can be inferred from the photos, (2) we compared the human and machine predictions on the same set of photos, and (3) we tested how the human prediction accuracy changes if we show individuals more training photos and we found that the accuracy does not increase dramatically (Study 2).

### Future work

This work opens many promising directions for future research. Here we highlight a few. First, we would like to further explore how the human and the machine predictions can be combined to improve their individual levels of accuracy. As we observed in “Machine vs. human predictions”, in more than one-third of all photos only the human or only the machine predictions were correct. We imagine two ways of combining humans and machines: (1) extending the feature representation of the machine learning models to include the human predictions, along with annotator’s confidence and prior accuracy rate, or (2) enriching the photos shown to the human annotators by overlaying information related to the group characteristics that the machine learning algorithm deemed relevant for its prediction.

Second, in this work, we decided to use traditional, off-the-shelf machine learning approaches. More recent methods, such as Convolutional Neural Networks (CNNs), have shown a remarkable improvement in accuracy on a number of computer vision tasks^43^. There are two key reasons why we refrained from using CNNs in this project: (1) the internal feature representations learned by these models are not interpretable and are hard to relate to the characteristics of the group in the photo, and (2) these models can sometimes capture artifacts in the photos that are predictive of the outcome, but are not substantively relevant to the classification problem (e.g., distinguishing between images of dogs versus wolves by focusing on the background, grass vs. snow, rather than on the animal itself^44^.) However, if the sole goal of building a model is prediction, without a need for interpretability, then CNNs might be a better approach.

## Ethical concerns and reproducibility

This study was approved by the Institution Review Board (IRB) at the Massachusetts Institute of Technology (MIT) and was considered minimal risk. All methods were carried out in accordance with relevant guidelines and regulations. Informed consent was obtained from all subjects in Study 1 and Study 2. All photos used in this work are publicly available (“Data collection”).

In this research, we performed analysis of thousands of group photos to infer what factors, revealed by these photos, predict success. Our analysis was done at the aggregate level, and our goals and procedures are ethically defensible. These goals were to (1) highlight the capabilities and limitations of current off-the-shelf algorithms on this task, (2) how they compare to humans doing the same task, (3) whether humans can increase their prediction success by training, and (4) identifying group characteristics that correlate with successful groups. While working towards these goals we highlighted some group features that can be recovered from their photos. The purpose of this is to understand and explain the mechanisms of the decision making both by humans and machines, and therefore why the findings of (1), (2), and (3) are sensible (i.e., why the prediction works), rather than to provide suggestions on how to use a group photo to make decisions on hiring or excluding individuals from a group.

Nevertheless, making inference of success (or failure) based on a photo is an ethically complicated, questionable and possibly problematic issue in general. This is manifested by the increasing controversy around the use of facial recognition technology to make decisions with high-stake consequences on individuals (e.g., identifying potential criminals)^45^, as well as the use of available social media information to predict personal traits^46^ or job prospects^47^, following the seminal work on algorithmic discrimination^48–50^. The fact that our goals for performing this research are ethically justifiable does not wholly shield this work from contributing to unethical practices. Therefore, we emphasize here that the data collected and the findings from this research should not be used to justify: (1) making hiring decisions based on someone’s appearance or use it to assess their potential contribution to group success, (2) assuming potential success (or failure) of a given group of individuals based on the personal or physical features of their members (or using their photos), or (3) deciding how to assemble teams of individuals based on their visual appearance or features (except when these features are directly related to the tasks e.g, height for basketball players).

## Conclusion

In this work, we studied the patterns of group performance behind Escape The Room with the aim to uncover what are the characteristics of successful groups and determine how accurately can humans and machines predict whether a group was successful. We find that larger, older, and gender diverse groups are more likely to escape. We also find that the best performing machine learning algorithm is better than human non-experts at spotting successful groups, even though human prediction accuracy significantly improves with training.

## Data Availability

The full dataset including aggregated features of each group of the $>43\hbox {K}$ groups used in our analyses is available at the following link: 10.7910/DVN/HDT2RN. All photos used in this work are publicly available, posted on public Facebook pages. However, we do not release the raw images, or the individual-level raw features extracted using the Face++ API. More details are provided at the link above.

## Abbreviations

API: Application programming interfaces
IQ: Intelligence quotient
OCR: Optical character recognition
URL: Uniform resource locator
SVM: Support vector machine
AUC: Area under curve
ROC: Receiver operating characteristics
CI: Confidence interval
CNN: Convolutional neural network
IRB: Institutional review board

## References

[1] Jones BF, Wuchty S, Uzzi B (2008). Multi-university research teams: Shifting impact, geography, and stratification in science. Science.

[2] Saavedra S, Hagerty K, Uzzi B (2011). Synchronicity, instant messaging, and performance among financial traders. Proc. Natl. Acad. Sci..

[3] Nielsen M (2020). Reinventing discovery: The new era of networked science.

[4] Mao, A., Mason, W., Suri, S. & Watts, D. J. An experimental study of team size and performance on a complex task. *PloS one* **11**, (2016). 10.1371/journal.pone.0153048 PMC4833429 27082239

[5] Azoulay P, Graff Zivin JS, Wang J (2010). Superstar extinction. Q. J. Econ..

[6] Pickard G (2011). Time-critical social mobilization. Science.

[7] Kittur, A. *et al.* The future of crowd work. In *Proceedings of the 2013 conference on Computer supported cooperative work*, 1301–1318 (2013).

[8] Howe J (2006). The rise of crowdsourcing. Wired Mag..

[9] Pescetelli N, Cebrian M, Rahwan I (2020). Beeme: Real-time internet control of situated human agents. Computer.

[10] Rutherford, A., Cebrian, M., Hong, I. & Rahwan, I. Impossible by conventional means: Ten years on from the darpa red balloon challenge. arXiv preprint arXiv:2008.05940 (2020).

[11] Belbin, R. M. *Management teams* (Routledge, 2010).

[12] Boxall, P. & Purcell, J. *Strategy and human resource management* (Macmillan International Higher Education, 2011).

[13] Frey CB, Osborne MA (2017). The future of employment: How susceptible are jobs to computerisation?. Technol. Forecast. Soc. Change.

[14] Lazer D, Brewer D, Christakis N, Fowler J, King G (2009). Life in the network: The coming age of computational social. Science.

[15] Woolley AW, Chabris CF, Pentland A, Hashmi N, Malone TW (2010). Evidence for a collective intelligence factor in the performance of human groups. Science.

[16] Hong L, Page SE (2004). Groups of diverse problem solvers can outperform groups of high-ability problem solvers. Proc. Natl. Acad. Sci..

[17] Østergaard CR, Timmermans B, Kristinsson K (2011). Does a different view create something new? the effect of employee diversity on innovation. Res. Policy.

[18] Stahl GK, Maznevski ML, Voigt A, Jonsen K (2010). Unraveling the effects of cultural diversity in teams: A meta-analysis of research on multicultural work groups. J. Int. Bus. Stud..

[19] Woolley AW, Aggarwal I, Malone TW (2015). Collective intelligence and group performance. Curr. Direct. Psychol. Sci..

[20] Fortunato S (2018). Science of science. Science.

[21] AlShebli BK, Rahwan T, Woon WL (2018). The preeminence of ethnic diversity in scientific collaboration. Nat. Commun..

[22] Nielsen MW (2017). Opinion: Gender diversity leads to better science. Proc. Natl. Acad. Sci..

[23] Jones BF, Weinberg BA (2011). Age dynamics in scientific creativity. Proc. Natl. Acad. Sci..

[24] McNeill, D. *The face: a natural history* (Back Bay Books, 2000).

[25] Oosterhof NN, Todorov A (2008). The functional basis of face evaluation. Proc. Natl. Acad. Sci..

[26] Yang D, Qi S, Ding C, Song Y (2011). An erp study on the time course of facial trustworthiness appraisal. Neurosci. Lett..

[27] Bonnefon J-F, Hopfensitz A, De Neys W (2013). The modular nature of trustworthiness detection. J. Exp. Psychol. Gen..

[28] Willis J, Todorov A (2006). First impressions: Making up your mind after a 100-ms exposure to a face. Psychol. Sci..

[29] Bar M, Neta M, Linz H (2006). Very first impressions. Emotion.

[30] Little AC, Burriss RP, Jones BC, Roberts SC (2007). Facial appearance affects voting decisions. Evol. Hum. Behav..

[31] Ballew CC, Todorov A (2007). Predicting political elections from rapid and unreflective face judgments. Proc. Natl. Acad. Sci..

[32] Graham, L. T. & Gosling, S. D. Can the ambiance of a place be determined by the user profiles of the people who visit it? In *Fifth International AAAI Conference on Weblogs and Social Media* (2011).

[33] Redi, M., Quercia, D., Graham, L. & Gosling, S. Like partying? your face says it all. predicting the ambiance of places with profile pictures. In *Ninth International AAAI Conference on Web and Social Media* (2015).

[34] Campbell M, Hoane AJ, Hsu F-H (2002). Deep blue. Artif. Intell..

[35] Schaeffer J (2007). Checkers is solved. Science.

[36] Ferrucci D (2010). Building watson: An overview of the deepqa project. AI Mag..

[37] Silver D (2016). Mastering the game of go with deep neural networks and tree search. Nature.

[38] Silver D (2018). A general reinforcement learning algorithm that masters chess, shogi, and go through self-play. Science.

[39] Jung, S.-G., An, J., Kwak, H., Salminen, J. & Jansen, B. J. Assessing the accuracy of four popular face recognition tools for inferring gender, age, and race. In *International Conference on Weblogs and Social Media*, 624–627, (2018).

[40] King, G., Tomz, M. & Wittenberg, J. Making the most of statistical analyses: Improving interpretation and presentation. *Am. J. Polit. Sci.*, 347–361, (2000).

[41] Varma S, Simon R (2006). Bias in error estimation when using cross-validation for model selection. BMC Bioinf..

[42] Demšar J (2006). Statistical comparisons of classifiers over multiple data sets. J. Mach. Learn. Res..

[43] LeCun Y, Bengio Y, Hinton G (2015). Deep learning. Nature.

[44] Ribeiro, M. T., Singh, S. & Guestrin, C. Why should i trust you? explaining the predictions of any classifier. In *Proceedings of the 22nd ACM SIGKDD international conference on knowledge discovery and data mining*, 1135–1144 (2016).

[45] Tidman, Z. Controversial facial recognition returns to scan faces in central london. *The Independent* (2020).

[46] Matz SC, Kosinski M, Nave G, Stillwell DJ (2017). Psychological targeting as an effective approach to digital mass persuasion. Proc. Natl. Acad. Sci..

[47] Kern ML, McCarthy PX, Chakrabarty D, Rizoiu M-A (2019). Social media-predicted personality traits and values can help match people to their ideal jobs. Proc. Natl. Acad. Sci..

[48] Buolamwini, J. & Gebru, T. Gender shades: Intersectional accuracy disparities in commercial gender classification. In *Conference on fairness, accountability and transparency*, 77–91 (2018).

[49] Raji, I. D. & Buolamwini, J. Actionable auditing: Investigating the impact of publicly naming biased performance results of commercial ai products. In *Proceedings of the 2019 AAAI/ACM Conference on AI, Ethics, and Society*, 429–435 (2019).

[50] Raji, I. D. *et al.* Saving face: Investigating the ethical concerns of facial recognition auditing. In *Proceedings of the AAAI/ACM Conference on AI, Ethics, and Society*, 145–151 (2020).

