# Publisher Correction: Algorithmic and human prediction of success in human collaboration from visual features

**DOI:** 10.1038/s41598-021-83429-0

**Published:** 2021-02-08

**Authors:** Martin Saveski, Edmond Awad, Iyad Rahwan, Manuel Cebrian

**Affiliations:** 1 Media Lab, Massachusetts Institute of Technology, Cambridge, MA USA; 2 Department of Economics, University of Exeter Business School, Exeter, UK; 3 Centre for Humans and Machines, Max-Planck Institute for Human Development, Berlin, Germany

Correction to: *Scientific Reports* 10.1038/s41598-021-81145-3, published online 02 February 2021

The original version of this Article omitted an affiliation for Edmond Awad. The correct affiliations for Edmond Awad are listed below:

Department of Economics, University of Exeter Business School, Exeter, UK

Centre for Humans and Machines, Max-Planck Institute for Human Development, Berlin, Germany

This error has now been corrected in the PDF and HTML versions of the Article.

